# Unmasking preclinical Alzheimer's pathology: auditory distraction cost and occupational reserve jointly discriminate amyloid positivity in subjective cognitive decline

**DOI:** 10.1002/alz.71874

**Published:** 2026-09-21

**Authors:** Yating Wang, Yuanhong Chen, Haifan Kong, Weineng Chen, Fengjuan Su, Yifan Zheng, Yingying Fang, Lishan Lin, Jiayi Zhou, Yucheng Li, Ya Zhang, Anqi Huang, Michael F. Bergeron, James O. Clifford Jr, J Wesson Ashford, Xianbo Zhou, Zhongliang Dai, Wenli Sheng, Zhong Pei

**Affiliations:** ^1^ Department of Neurology, Guangdong Provincial Key Laboratory of Diagnosis and Treatment of Major Neurological Diseases National Key Clinical Department and Key Discipline of Neurology, The First Affiliated Hospital, Sun Yat‐sen University Guangzhou China; ^2^ Department of Health Sciences University of Hartford West Hartford Connecticut USA; ^3^ Department of Psychology College of San Mateo San Mateo California USA; ^4^ War Related Illness and Injury Study Center (WRIISC) VA Palo Alto Health Care System Palo Alto California USA; ^5^ Department of Psychiatry & Behavioral Sciences Stanford University Palo Alto California USA; ^6^ War Related Illness & Injury Study Center VA Palo Alto Health Care System Palo Alto California USA; ^7^ Center for Alzheimer's Research Washington Institute of Clinical Research Vienna Virginia USA; ^8^ AstraNeura, Co., Ltd. Shanghai China; ^9^ Department of Anesthesiology, Shenzhen People's Hospital (The First Affliated Hospital Southern University of Science and Technology) Shenzhen China; ^10^ Department of Anesthesiology, The Second Clinical Medical College Jinan University Shenzhen China

**Keywords:** Alzheimer's disease, cognitive reserve, distraction cost, MemTrax, subjective cognitive decline

## Abstract

**INTRODUCTION:**

Conventional cognitive instruments exhibit limited efficacy in detecting insidious cognitive decline in subjective cognitive decline (SCD) due to neural compensation. We hypothesized that auditory distraction during a visual memory task would unmask preclinical amyloid beta (Aβ) vulnerability.

**METHODS:**

We evaluated 112 participants (SCD and mild cognitive impairment [MCI]) using the MemTrax test under quiet and noisy conditions to quantify distraction costs (d‐MTxc). Aβ status and cognitive reserve were assessed via positron emission tomography and the Cognitive Reserve Index questionnaire (CRIq).

**RESULTS:**

Aβ‐positive SCD individuals performed normally in quiet but showed higher d‐MTxc than Aβ‐negative peers (11.17 ± 5.762 vs 6.67 ± 5.096). Higher CRIq‐WorkingActivity (CRIW) scores identified Aβ positivity and moderated performance. The combination of d‐MTxc and CRIW accurately classified Aβ positivity (AUC = 0.836), whereas MCI individuals exhibited metabolic network fragmentation.

**DISCUSSION:**

Auditory distraction taxes cognitive capacity to expose compensatory failures coupled with Aβ burden. This paradigm, interacting with occupational reserve, offers a promising digital biomarker framework for preclinical Alzheimer's screening.

## Highlights

- The d‐MTxc metric quantifies heightened distraction costs in Aβ‐positive individuals with subjective cognitive decline under a cross‐modal cognitive stress test.
- Integrating dynamic distraction cost (d‐MTxc) with static occupational cognitive reserve (CRIW) establishes a brief, accessible framework for screening Aβ pathology in the SCD population.
- Elevated occupational reserve is associated with intact daily functioning in Aβ‐positive SCD despite greater amyloid burden and regional hypometabolism, consistent with a compensatory mechanism.
- This brief, non‐invasive cognitive stress test provides a scalable cost‐effective strategy for large‐scale community screening of preclinical AD.

## 1 BACKGROUND

Alzheimer's disease pathology accumulates for decades prior to the onset of dementia.^1^ Subjective cognitive decline (SCD) denotes a self‐reported awareness of decline in cognitive abilities despite normal performance on objective tests.^2^ SCD is typically assessed using standardized questionnaires (e.g., the SCD Questionnaire‐9).^3^ It is this capacity for self‐appraisal, rather than objective psychometric performance, that may offer a unique avenue for detecting the prodromal stages of dementia. Assessing the subtle changes during the preclinical stage, before they manifest as observable behavioral declines, may facilitate early therapeutic intervention. The SCD Initiative consensus identifies preclinical SCD as a critical risk factor for progression to mild cognitive impairment (MCI) and dementia.^2^ However, identifying objective measures of Alzheimer's disease‐related SCD is challenging. Current screening tools – including the Mini‐Mental State Examination (MMSE) and the Montreal Cognitive Assessment (MoCA) and emerging digital biomarkers like MemTrax – lack the sensitivity to detect subtle deficits in this population.^4^ Lack of sensitivity may indicate that cognitive reserve (CR) masks the early symptoms caused by pathology. While these tools can distinguish MCI from healthy controls,^4^, ^5^ the period between SCD and MCI remains a diagnostic “blind‐spot”.^6^, ^7^, ^8^ Even with SCD questionnaires, self‐reported measures fail to distinguish between Alzheimer's disease‐related SCD and normal aging.^9^, ^10^ Patients frequently present with memory complaints but score within normal ranges on objective tests, making the diagnosis unduly subjective and thus leaving the etiology of their symptoms unresolved.

**RESEARCH IN CONTEXT**

1. **Systematic review**: SCD is a key preclinical Alzheimer's disease state. Standard neuropsychological and conventional MemTrax assessments cannot detect subtle Aβ‐related deficits in SCD, as CR masks underlying Alzheimer's disease pathology, creating an unmet need for scalable early screening tools.
2. **Interpretation**: This study validated AD‐MemTrax, a cognitive stress test using auditory interference to break neural compensation. While Aβ‐positive and negative SCD showed identical baseline performance, Aβ‐positive participants had larger distraction‐induced memory declines (d‐MTxc). Combining d‐MTxc with CRIW achieved strong Aβ diagnostic accuracy (raw AUC = 0.836, corrected AUC = 0.826). Neuroimaging revealed divergent mechanisms: CRIW reflected long‐term tolerance to default mode network hypometabolism, whereas d‐MTxc captured transient network breakdown under cognitive load. High occupational reserve sustains normal daily function despite heavy Aβ burden, proving the two markers complement each other.
3. **Future directions**: Large community prospective trials, multi‐ethnic replications, long‐term longitudinal follow‐ups, auditory/visual distractor comparisons, and head‐to‐head tests with plasma pTau217 are required to refine a tiered preclinical Alzheimer's disease screening pipeline for clinical translation.

Emerging evidence suggests that while global cognition appears largely preserved in SCD, specific domains such as attention and executive control may be compromised.^11^, ^12^ Attentional complaints in SCD have been linked to amyloid positivity.^13^ Consistent with this, performance on attentional control tasks (e.g., the Stroop test) correlates with functional connectivity in the Default Mode Network (DMN).^14^ However, most conventional cognitive assessments measure performance under optimal, quiet conditions, which may allow compensatory mechanisms to mask underlying deficits. Cognitive compensation allows high‐reserve individuals to sustain cognitive homeostasis and mask underlying deficits.^15^ In this case, early changes in adaptive networks help sustain cognitive performance. However, this compensation is finite; once reserve capacity is exceeded, cognitive decline becomes apparent.^4^, ^15^ To unmask these preclinical deficits, we propose a “cognitive stress test”^16^ utilizing cross‐modal interference. Given the established link between auditory distraction and attention,^17^ we hypothesized that, while standard visual tasks are generally manageable for SCD patients, introducing targeted distractions during cognitive assessments – such as auditory interference during a visual task – would noticeably challenge the emerging state of compromised brain function.

MemTrax is an online cognitive assessment tool based on a continuous recognition task that allows for repeated measurements.^18^ It specifically activates the temporo‐parietal regions of the neocortex and the hippocampus – regions associated with memory encoding that are susceptible to early amyloid beta (Aβ) deposition and structural damage.^19^, ^20^, ^21^, ^22^ Therefore, performance on MemTrax is highly sensitive to the efficiency of memory encoding.^23^ However, under standard quiet testing conditions, cognitive compensation may mask underlying impairments. To unmask this cognitive vulnerability, we introduced an auditory distraction. Noise increases the neural burden and the resource demand required for the brain to accomplish task demands,^17^ forcing a reallocation of limited cognitive resources. We hypothesized that this visual‐auditory cross‐modal interference would disproportionately affect and overwhelm the already compromised neural systems in Aβ‐positive individuals with SCD.

To test this hypothesis, MemTrax was performed in a cross‐modal paradigm with auditory distraction. By quantifying the “distraction cost” (d‐MTxc) – the performance decrement induced by auditory noise – we aim to measure the fragility of cognitive compensation. Furthermore, considering the important compensatory role of CR in Alzheimer's disease‐related pathology, we used the Cognitive Reserve Index questionnaire (CRIq) to quantify CR and investigated its role – specifically occupational complexity – in moderating the relationship between Aβ pathology and cognitive performance at the SCD stage of Alzheimer's disease.^24^ The present study may help elucidate the neurocognitive mechanisms underlying preclinical Alzheimer's disease and inform early intervention strategies.

## 2 METHODS

### 2.1 Participants

One hundred twelve participants were recruited from the Guangzhou Healthy Aging and Dementia Cohort between May 2024 and March 2026. The sample consisted of 49 males and 63 females, with a median age of 68 years (range: 50 to 86 years). Participants underwent a comprehensive evaluation including the MMSE, MoCA, Clinical Dementia Rating (CDR), CRIq, and the MemTrax Continuous Recognition Test, all administered within 1 week prior to positron emission tomography (PET)/computed tomography (CT) imaging with 18F‐Florbetapir for Aβ deposition and 18F‐fluorodeoxyglucose (FDG) to assess regional cerebral glucose metabolism. Inclusion criteria included age ≥ 50 years, ongoing memory complaints, CDR ≤ 0.5, and MMSE score ≥ 20.^25^ Participants were defined and stratified based on Aβ status, in conjunction with their performance on the MMSE and MemTrax: (1) SCD group: defined by MMSE ≥ 25^26^, ^27^ and MemTrax accuracy ≥ 80% (based on established cutoffs for distinguishing MCI‐Alzheimer's disease from healthy controls from our earlier study)^5^; (2) MCI group: Participants who did not meet the SCD criteria, presenting with objective cognitive impairment as evidenced by either MMSE < 25 or MemTrax accuracy < 80%, while maintaining functional independence.

### 2.2 MemTrax and auditory distraction protocol

The detailed description and theoretical background of MemTrax have been published elsewhere.^23^ Briefly, during the assessment, participants are presented with 50 visual stimuli (images) and instructed to attend to each stimulus while responding as quickly as possible upon detecting a repeated image. Each image during this Continuous Recognition Test is displayed for up to 3 s or until a response is given, taking approximately 90 s in total to complete.^28^

In this study, the MemTrax Continuous Recognition Test was administered under two conditions to assess susceptibility to auditory interference. Under the quiet condition, participants completed the test in a clinical room with ambient background noise maintained at 45 to 50 dB. The accuracy score, expressed as the percentage correct (range 0% to 100%), was recorded as Q‐MTxc. To prevent potential carryover effects of auditory distraction from confounding baseline performance, the quiet condition was consistently administered first, with the brief duration and randomized item sequences of the MemTrax test minimizing potential practice effects. Following a 2‐min rest, participants underwent retesting under the noisy condition, where they performed the test while exposed to recorded street construction and traffic noise (65 to 70 dB). This range was selected to induce cognitive interference without triggering physiological stress responses in older adults: noise below ∼55 dB maintains acoustic comfort, while levels exceeding ∼70 dB can elicit cardiovascular stress responses (e.g., altered heart rate variability, increased electrodermal activity).^29^ The recorded street construction and traffic noise was first digitally standardized in Audacity by scaling the root‐mean‐square (RMS) amplitude to −20 dBFS, establishing a uniform energy baseline while preserving the natural temporal and spectral fluctuations. During the experiment, this same audio file was delivered to all participants via a fixed external speaker. Physical output volume was calibrated prior to testing using a sound level meter at the participant's ear level. This maintained the final auditory stimulus at an average equivalent continuous sound level (L_Aeq_) of approximately 67 dB, with natural dynamic fluctuations confined within the 65‐ to 70‐dB range to ensure uniform stimulation across the cohort. The accuracy score was similarly recorded as a percentage, denoted by N‐MTxc. This sound pressure level mimics typical urban environments and remains well below occupational safety standards (85 dB for 8 h), thereby increasing cognitive load without inducing a startle response or hearing risk. The distraction cost (d‐MTxc) was defined as the absolute decline in performance under distraction and calculated as

d−MTxc=Q−MTxc−N−MTxc.

Since both Q‐MTxc and N‐MTxc are percentages, d‐MTxc is expressed in percentage points (pp). Mean reaction times (RTs) were similarly recorded for both conditions, with values expressed in seconds. Similarly, the reaction‐time cost (d‐MTxrt) was calculated as Q‐MTxrt minus N‐MTxrt, with d‐MTxrt expressed in seconds.

### 2.3 CR assessment

CR was quantified using the CRIq.^30^ This instrument is designed to measure CR and generates three subscores: (1) CRI‐Education (CRIE): based on years of education and training; (2) CRI‐Working Activity (CRIW): based on the cognitive complexity and responsibility level of individuals' occupations; and (3) CRI‐Leisure (CRIL): based on the frequency of intellectual, social, and physical activities.

### 2.4 PET data acquisition and analysis

Aβ status (positive/negative) was determined via visual interpretation of 18F‐Florbetapir PET scans by two independent neuroradiologists. All PET images were spatially normalized to Montreal Neurological Institute space using an adaptive probabilistic brain atlas‐based method.^31^ For quantitative analysis, standardized uptake value ratios (SUVRs) were calculated using the whole cerebellum as the reference region. The SUVR values reflecting Aβ deposition/FDG were then extracted from 90 volumes of interest (VOIs) based on the Automated Anatomical Labeling (AAL) atlas. A group‐based, weighted, undirected FDG metabolic network matrix was constructed using GRETNA by calculating the Pearson correlation coefficient between each pair of VOIs.^32^

### 2.5 Statistical analysis

Statistical analyses were performed using SPSS 21.0 and R software. Variables following a normal distribution are reported as mean ± SD and were analyzed using independent samples *t*‐tests, whereas non‐normally distributed variables are reported as median (interquartile range: P25 to P75) and analyzed using Mann‐Whitney U tests. Differences in categorical variables were evaluated with chi‐squared tests. Binary logistic regression determined independent predictors of Aβ positivity, and receiver operating characteristic (ROC) analysis evaluated predictive utility. To evaluate model stability and mitigate the risk of overfitting, bootstrap internal validation was performed using 1000 resamples. The optimism‐corrected area under the curve (AUC), calibration slope, mean absolute error, and Brier score were calculated to assess the discrimination and calibration of the probability estimates. Correlation analysis between variables was performed using Pearson or Spearman rank correlation methods. Significance was set at *p* < 0.05.

## 3 RESULTS

### 3.1 Auditory distraction cost (d‐MTxc) and occupational cognitive reserve (CRIW) jointly identify Aβ‐positive status in SCD

In the SCD cohort (*n* = 65; Aβ‐negative *n* = 24, Aβ‐positive *n* = 41), demographic variables (age, sex) and standard cognitive scores (MoCA) did not differ significantly between Aβ‐negative and Aβ‐positive groups (all *p* > 0.05). Notably, MemTrax accuracy under quiet conditions (Q‐MTxc) was similar between the Aβ‐negative group and the Aβ‐positive group (88.25% vs 88.34%, *p* = 0.943). However, the introduction of an auditory distraction revealed a distinct cognitive deficit in the Aβ‐positive group. Their performance accuracy under noise (N‐MTxc) declined significantly to 77.17%, compared to 81.58% in the Aβ‐negative group (*p* = 0.021). Consequently, the calculated distraction cost (d‐MTxc) was significantly higher in Aβ‐positive subjects (11.17 ± 5.762 pp) compared to Aβ‐negative subjects (6.67 ± 5.096 pp, *p* = 0.002). In contrast, reaction times under both quiet and noisy conditions, as well as the reaction‐time cost (d‐MTxrt), showed no significant differences between Aβ‐positive and Aβ‐negative SCD groups (Table 1). To ensure this finding was not confounded by subtle baseline imbalances, a sensitivity analysis using an analysis of covariance (ANCOVA) was performed, with d‐MTxc as the dependent variable, Aβ status as the independent variable, and sex and baseline MoCA scores as covariates. The results demonstrated that, even after controlling for sex and baseline MoCA scores as covariates, the adjusted mean difference in d‐MTxc between the Aβ‐negative and Aβ‐positive groups remained robust (*F* = 11.775, *p* = 0.001). Furthermore, neither sex (*p* = 0.120) nor MoCA (*p* = 0.657) emerged as a significant factor influencing d‐MTxc, indicating that the observed effect was independent of demographic and baseline cognitive variations.

**TABLE 1:** Demographic, clinical characteristics, and cognitive reserve level of SCD participants.

	Aβ‐negative (*n* = 24)	Aβ‐positive (*n* = 41)	χ^2^/t/z	*p* value
Age, mean (SD), years	64.13 (9.368)	66.98 (8.054)	−1.296	0.2
Sex, female, No. (%)	17 (70.8)	19 (46.3)	3.675	0.055
MoCA, mean (SD)	24.38 (3.090)	22.93 (3.020)	1.85	0.069
Q‐MTxc, mean (SD), %	88.25 (4.306)	88.34 (5.252)	−0.072	0.943
Q‐MTxrt, mean (SD), s	1.251 (0.243)	1.266 (0.258)	−0.226	0.822
N‐MTxc, mean (SD), %	81.58 (6.213)	77.17 (7.823)	2.359	**0.021**
N‐MTxrt, mean (SD), s	1.216 (0.271)	1.245 (0.276)	−0.41	0.683
d‐MTxc, mean (SD), pp	6.67 (5.096)	11.17 (5.762)	−3.17	**0.002**
d‐MTxrt, median [P25, P75], s	0.044 [−0.047, 0.161]	0.069 [−0.095, 0.130]	−0.306	0.76
CRIE, mean (SD)	107.25 (16.12)	110.98 (11.337)	−1.091	0.279
CRIW, mean (SD)	107.83 (12.896)	122.41 (13.769)	−4.216	**<0.001**
CRIL, mean (SD)	96.75 (14.195)	99.95 (11.333)	−1	0.321
CRIT, mean (SD)	105.17 (16.306)	114.78 (10.572)	−2.886	**0.005**

*Note*: χ^2^ values are reported for categorical variables (chi‐squared test); *t* values are reported for normally distributed continuous variables (independent samples *t*‐test); z values are reported for non‐normally distributed continuous variables (Mann‐Whitney U test).

Abbreviation: CRIE, education scores of cognitive reserve index questionnaire; CRIL, leisure activity scores of cognitive reserve index questionnaire; CRIT, total scores of cognitive reserve index questionnaire; CRIW, work scores of cognitive reserve index questionnaire; d‐MTxc, distraction cost representing difference in MemTrax accuracy between the quiet and noisy environments; d‐MTxrt, the difference in MemTrax reaction time between the quiet and noisy environments; MoCA, Montreal Cognitive Assessment; N‐MTxc, accuracy rate of MemTrax in a noisy environment; N‐MTxrt, reaction time of MemTrax in a noisy environment; Q‐MTxc, accuracy rate of MemTrax in a quiet environment; Q‐MTxrt, reaction time of MemTrax in a quiet environment; pp, percentage point.

**FIGURE 1:**
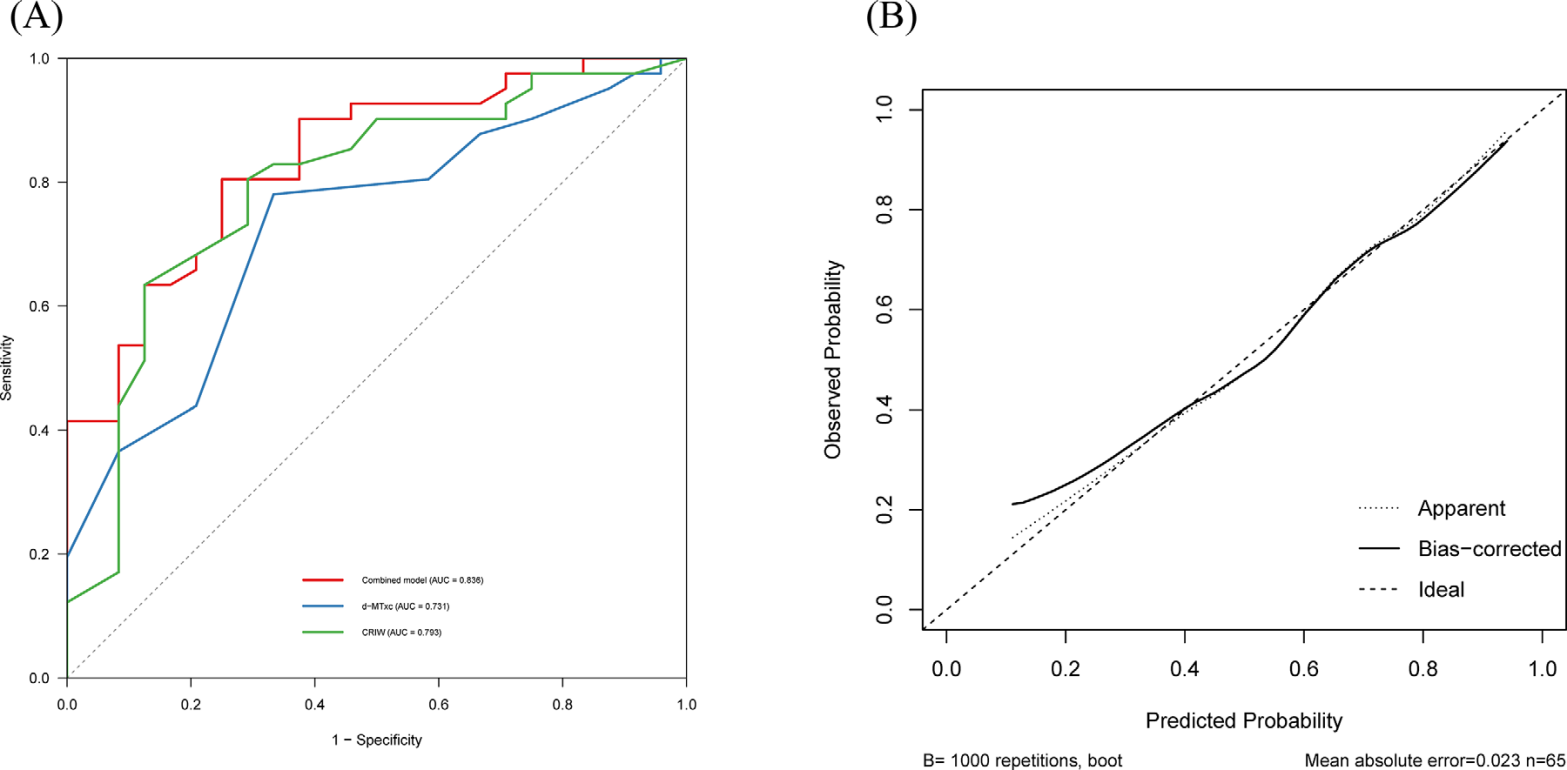
(A) ROC analysis of combination of independent predictors. (B) Calibration curve.

Analysis of CRIq subscores revealed that while education (CRIE) and leisure (CRIL) scores were comparable, the Aβ‐positive SCD group possessed significantly higher CRIW compared to the Aβ‐negative SCD group (122.41 vs 107.83, *p* < 0.001) (Table 1). Variables showing statistically significant inter‐group differences in univariate analysis (Table 1) were entered into a binary logistic regression using the forward Wald method. Logistic regression identified both d‐MTxc (odds ratio [OR]: 1.158, 95% confidence interval [CI]: 1.024 to 1.31) and CRIW (OR: 1.082, 95% CI: 1.03 to 1.136) as independent classifiers of Aβ‐positive status (Table 2). A combined predictive model utilizing both metrics achieved an AUC of 0.836 (sensitivity 80.5%, specificity 75%) (Figure 1A). Bootstrap internal validation (B = 1000 resamples) confirmed model stability. The optimism‐corrected AUC was 0.826 (95% CI: 0.742 to 0.910), with an optimism of 0.010 relative to the apparent AUC of 0.836, indicating limited overfitting. The model yielded a low Brier score of 0.134, demonstrating reliable probability estimates. Furthermore, the corrected calibration slope was 0.923, and the bootstrap‐corrected calibration curve showed close alignment between predicted and observed Aβ positivity probabilities (mean absolute error = 0.023; Figure 1B).

**TABLE 2:** Independent factors predicting Aβ positivity in SCD participants.

	B	Wald χ^2^	*p*	OR	95% CI
d‐MTxc	0.147	5.44	0.02	1.158	1.024 to 1.31
CRIW	0.079	9.991	0.002	1.082	1.03 to 1.136

Abbreviations: CI, confidence interval; CRIW, work scores of cognitive reserve index questionnaire; d‐MTxc, distraction cost representing difference in MemTrax accuracy between quiet and noisy environments; OR; odds ratio.

### 3.2 Distinctive patterns of distraction vulnerability in Aβ‐positive SCD versus MCI

Given the comparatively small Aβ‐negative MCI sample resulting from the high prevalence of amyloid pathology, direct comparisons between MCI subgroups would lack statistical power and carry a high risk of confounding by non‐Alzheimer's disease pathologies. Nonetheless, for completeness, MemTrax performance comparisons between MCI subgroups are provided in Table S1.

Compared to Aβ‐positive SCD patients, the Aβ‐positive MCI subgroup exhibited global cognitive decline, as indicated by significantly lower MemTrax performance (Q‐MTxc, N‐MTxc) across all experimental conditions (Table 3). Notably, the distraction cost in the Aβ‐positive MCI subgroup (5.95 ± 7.564 pp) was significantly lower than that in the Aβ‐positive SCD subgroup (11.17 ± 5.762 pp, *p* = 0.001). Regarding CR, lower CRIW scores were observed in the Aβ‐positive MCI subgroup compared to the Aβ‐positive SCD subgroup (Table 3).

**TABLE 3:** Comparison of clinical data between Aβ‐positive SCD and MCI participants.

	Aβ‐positive SCD (*n* = 41)	Aβ‐positive MCI (*n* = 37)	*t*	*p*
Q‐MTxc, mean (SD), %	88.34 (5.252)	73.3 (8.289)	9.458	**<0.001**
N‐MTxc, mean (SD), %	77.17 (7.823)	67.41 (9.32)	5.028	**<0.001**
d‐MTxc, mean (SD), pp	11.17 (5.762)	5.95 (7.564)	3.404	**0.001**
CRIW, mean (SD)	122.41 (13.769)	113.22 (18.76)	2.446	**0.017**

*Note*: *t* values are reported for normally distributed continuous variables (independent samples *t*‐test).

Abbreviations: CRIW, work scores of cognitive reserve index questionnaire;N‐MTxc, accuracy rate of MemTrax in a noisy environment; pp, percentage point; d‐MTxc, the distraction cost representing the difference in MemTrax accuracy between the quiet and noisy environments; Q‐MTxc, accuracy rate of MemTrax in a quiet environment.

To further investigate the relationships among CR, distraction cost, and the manifestation of cognitive status (SCD vs MCI) in Aβ‐positive patients, we stratified the Aβ‐positive patients (SCD and MCI) into high‐ and low‐d‐MTxc subgroups based on the median d‐MTxc value of 10 pp derived from the 78 patients. The results revealed that the high‐d‐MTxc subgroup contained a higher proportion of SCD patients (66.7%), whereas the low‐d‐MTxc subgroup consisted predominantly of MCI patients (70%) (*p* = 0.002) (Table 4). Subsequent subgroup analysis of the 48 participants in the high‐d‐MTxc group demonstrated that Aβ‐positive SCD patients had significantly higher CRIW, CRIL, and CRIT scores than Aβ‐positive MCI patients, suggesting that higher occupational and total CR may play a compensatory role in mitigating Aβ‐associated cognitive decline in SCD (Table 5).

**TABLE 4:** Cognitive status of patients between high‐ and low‐distraction cost groups.

	low‐d‐MTxc (*n* = 30)	high‐d‐MTxc (*n* = 48)	χ^2^	*p* value
SCD, No. (%)	9 (30)	32 (66.7)	9.954	**0.002**
MCI, No. (%)	21 (70)	16 (33.3)		

Abbreviation: d‐MTxc, the distraction cost representing difference in MemTrax accuracy between quiet and noisy environments; MCI, mild cognitive impairment; SCD, subjective cognitive decline.

**TABLE 5:** Differences in distribution of CR between SCD and MCI groups among Aβ‐positive patients with high distraction cost.

	Aβ‐positive SCD (*n* = 32)	Aβ‐positive MCI (*n* = 16)	t	*p* value
CRIE, mean (SD)	111.97 (9.956)	105.13 (14.674)	1.91	0.062
CRIW, mean (SD)	122.81 (14.116)	109.25 (18.724)	2.809	**0.007**
CRIL, mean (SD)	98.75 (11.13)	91.13 (10.651)	2.269	**0.028**
CRIT, mean (SD)	114.84 (9.775)	102.25 (15.524)	2.964	**0.007**

*Note*: *t* values are reported for normally distributed continuous variables (independent samples *t*‐test).

Abbreviations: CRIE, education scores of cognitive reserve index questionnaire; CRIL, leisure activity scores of cognitive reserve index questionnaire; CRIT, total scores of cognitive reserve index questionnaire; CRIW, work scores of cognitive reserve index questionnaire.

### 3.3 Neural mechanisms linking Aβ deposition, metabolism, distraction cost, and CR

Utilizing the 90 brain regions parcellated by the AAL template, we initially explored the associations between regional Aβ SUVRs and both d‐MTxc and CRIW scores. Preliminary bivariate analyses revealed consistent positive correlation trends, with 40 regions exhibiting significant correlations with both indices (uncorrected *p* < 0.05) (Figure S1).

**FIGURE 2:**
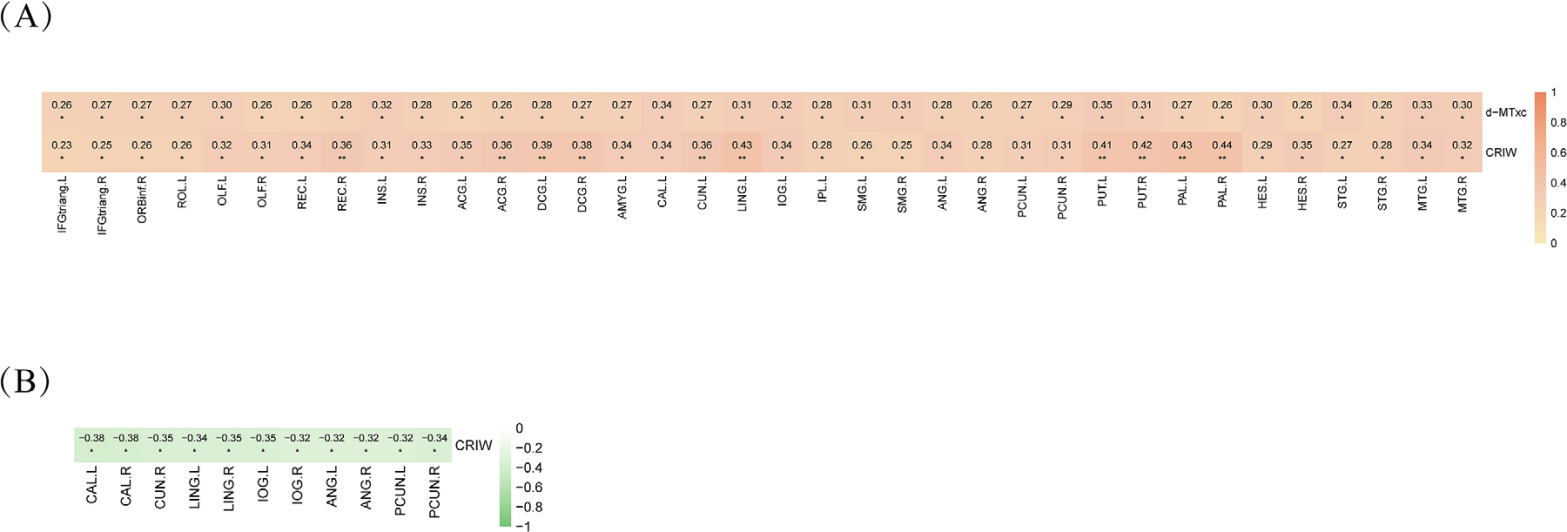
(A) Heatmap of FDR‐corrected regional correlations between Aβ SUVR and d‐MTxc/CRIW scores. The 36 brain regions displayed survived whole‐brain Benjamini‐Hochberg FDR correction, maintaining significant positive correlations with both clinical indices (both *q* < 0.05). (B) Heatmap of FDR‐corrected regional correlations between FDG SUVR and CRIW scores. The 11 brain regions displayed survived whole‐brain Benjamini‐Hochberg FDR correction, maintaining significant negative correlations with CRIW (*q* < 0.05). Numbers within cells represent correlation coefficients (Pearson's *r* or Spearman's *r_s_*, determined based on data normality). Asterisks denote FDR‐corrected significance levels: *q < 0.05, **q < 0.01, ***q < 0.001.

To formally validate these exploratory findings and control for multiple comparisons across all 90 region‐of‐interest (ROI)‐based tests, one‐sided correlation tests were subsequently performed with Benjamini‐Hochberg false discovery rate (FDR) correction. This analysis confirmed that 36 regions maintained significant positive correlations with both d‐MTxc and CRIW scores at the whole‐brain level (both *q* < 0.05) (Figure 2A). These identified regions include the insula, anterior cingulate, angular gyrus, and precuneus, among others; detailed regional information is provided in Table S2.

To further elucidate downstream metabolic mechanisms, we applied the same 90‐ROI correlation framework to regional FDG‐PET data. CRIW was negatively correlated with FDG SUVRs in 19 brain regions at an uncorrected threshold (Figure S2). No significant correlations were observed between d‐MTxc and regional FDG uptake. Following one‐sided testing with whole‐brain Benjamini‐Hochberg FDR correction (*q* < 0.05), CRIW maintained significant negative correlations with FDG uptake in 11 core regions (Figure 2B), prominently featuring the precuneus and angular gyrus (detailed regional information is provided in Table S3). Notably, no significant correlations were observed between d‐MTxc and regional FDG uptake across any ROIs.

### 3.4 Altered brain metabolic network connectivity from SCD to MCI

**FIGURE 3:**
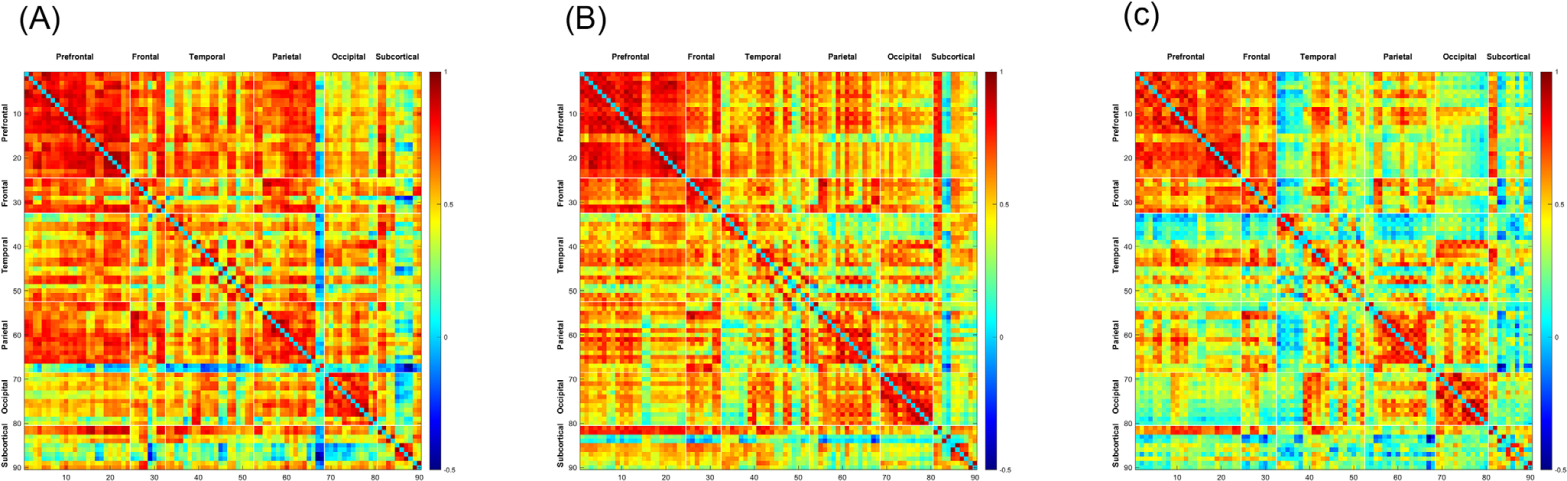
Distribution of 90‐region FDG matrix, organized by lobe, across the three groups. (A) SCD Aβ‐negative group. (B) SCD Aβ‐positive group. (C) MCI Aβ‐positive group.

Metabolic network connectivity analysis revealed that the Aβ‐positive MCI group (Figure 3C) exhibited weaker connectivity compared to both SCD groups (Aβ‐negative: Figure 3A; Aβ‐positive: Figure 3B), with the differences being particularly pronounced in the temporal, parietal, and occipital lobes (Figure 3). This indicates that the progression from SCD to MCI in Aβ‐positive patients may be accompanied by impaired metabolic network integrity in these brain regions, which may underlie the global cognitive decline observed in MCI.

## 4 DISCUSSION

This study establishes Auditory Distraction MemTrax (AD‐MemTrax) as a viable “cognitive stress test” for preclinical Alzheimer's disease detection at the SCD stage. By quantifying the performance gap between quiet and noisy conditions (d‐MTxc), combined with occupational cognitive reserve (CRIW), we accurately identified Aβ‐positive SCD individuals (AUC = 0.836). These findings demonstrate that integrated AD‐MemTrax and CR assessments enable non‐invasive, cost‐effective early Alzheimer's disease detection.

A major novel observation is that the auditory distraction cost effectively discriminates Aβ‐positive from Aβ‐negative individuals in SCD, a stage where standard neuropsychological tests remain uninformative. While age, sex, global cognition, and baseline MemTrax accuracy were comparable between Aβ subgroups, Aβ‐positive SCD subjects showed significantly greater accuracy decline under auditory distraction, resulting in higher d‐MTxc. This suggests that subtle cognitive alterations associated with early Alzheimer's disease pathology become apparent when the cognitive system is challenged by external interference.

The MemTrax paradigm engages multiple cognitive processes, including episodic memory encoding/retrieval, processing efficiency, and executive control.^18^ RTs did not differ between groups under either condition, ruling out generalized psychomotor slowing, motivational differences, or speed‐accuracy trade‐offs. Because d‐MTxc represents the intra‐individual performance decrement induced by elevated task demands, we propose it as a marker of cognitive inefficiency under increased task demands – a construct encompassing attentional resource allocation, executive control, and the ability to sustain mnemonic performance under competing neural resource demands. Cross‐modal stimulus presentation modulates attentional resource allocation,^33^ and prior research suggested that P300 amplitude reflected recruitment of attentional resources during effortful processing, with Alzheimer's disease patients exhibiting reduced P300 enhancement when additional resources are required.^34^, ^35^ We interpret these P300 findings as consistent with a general resource recruitment deficit rather than attentional impairment specifically; reduced P300 enhancement may equally reflect compromised working memory updating, context maintenance, or executive control under load. Taken together, these findings suggest that impaired cognitive compensation under neural resource constraint is consistent with the observed distraction cost.

Additionally, Aβ‐positive subjects exhibited significantly higher CRIW scores. This counterintuitive finding aligns with the CR threshold model: Individuals with greater occupational attainment can tolerate higher Aβ burden before objective impairment manifests.^36^ Higher occupational reserve may allow SCD individuals to sustain relatively normal function despite accumulating Aβ pathology, prolonging the SCD stage and delaying progression to MCI. This interpretation is reinforced by higher total CR in Aβ‐positive SCD, while education and leisure components did not differ – underscoring the specific contribution of lifelong occupational complexity.^37^

Furthermore, the observed association between higher CRIW and Aβ positivity may be interpreted in the context of collider stratification bias. Because our SCD cohort is defined by preserved objective cognition, individuals with both high amyloid burden and normal test performance are enriched for high CR. Lower‐reserve individuals with comparable pathology may have already progressed to MCI. Thus, the observed association could reflect CR compensation, collider stratification bias, or both. While these mechanisms are distinct – one biological and one statistical – they are not mutually exclusive and can produce observationally equivalent patterns in cross‐sectional data.

Importantly, our regional FDG‐PET analysis provides evidence consistent with the reserve interpretation. We observed significant negative correlation between CRIW and regional FDG metabolism within the SCD cohort (Figure 2B), such that higher occupational reserve was associated with greater cerebral hypometabolism in Alzheimer's disease‐vulnerable DMN regions (precuneus and angular gyrus). This suggests that higher‐reserve individuals sustain objective cognitive normality even with more pronounced neurodegeneration. While pure collider effects could theoretically induce spurious correlations, they would not inherently predict this regionally specific Alzheimer's disease‐vulnerability pattern. Longitudinal tracking is required to definitively determine whether high‐CRIW/Aβ‐positive SCD individuals exhibit differential cognitive trajectories compared with their low‐reserve peers.

Binary logistic regression confirmed that d‐MTxc and CRIW independently classified cerebral Aβ status, with their combined model achieving a superior AUC of 0.836. Integrating dynamic distractibility and static CR enhances preclinical Alzheimer's disease detection by identifying amyloid pathology masked by reserve compensation. Given the limited accessibility of amyloid imaging and the emerging adoption of blood‐based biomarkers, a tiered diagnostic workflow is essential. A brief cognitive stress test (AD‐MemTrax, ∼5  min) combined with reserve evaluation (CRIq, ∼5 min) provides a cost‐effective strategy for initial SCD screening in primary care, enabling targeted triaging of high‐risk individuals for confirmatory molecular testing. Furthermore, because molecular pathological burden does not strictly equate to functional impairment – as this relationship is heavily moderated by CR – AD‐MemTrax provides a functional dimension complementing the static ATN framework.

By the MCI stage, global cognitive decline was already established, and distraction cost no longer showed significant discriminatory value for Aβ status. These findings indicate that d‐MTxc is a stage‐sensitive marker most informative during preclinical SCD, when cognitive dysfunction is subtle and compensatory mechanisms remain active but vulnerable. Once clinical impairment emerges, the marker's discriminative capacity attenuates – likely because compensatory failure has become overt. Our data demonstrate higher d‐MTxc in Aβ‐positive SCD compared to Aβ‐positive MCI, but we cannot determine the full trajectory. Notably, we lack Aβ‐negative MCI data; this group is essential to distinguish whether attenuation reflects reserve depletion specifically or generalized impairment regardless of pathology. Longitudinal tracking is required to map the complete stage dependency of this marker.

Furthermore, the discriminative utility of d‐MTxc depends on CR heterogeneity within the sampled stage. In our SCD cohort, reserve variation created a detectable signal: High‐reserve individuals maintained objective cognition despite elevated distraction costs, while low‐reserve individuals with comparable pathology may have already progressed to MCI. In populations with uniformly low CR, the SCD window may be substantially narrowed. Our framework is therefore calibrated to the SCD stage with active but strained compensation; generalizability to low‐reserve populations requires prospective validation.

Further comparisons across diagnostic groups provided direct support for the compensatory role of CR. Aβ‐positive MCI participants showed significantly lower Q‐MTxc and N‐MTxc relative to Aβ‐positive SCD patients. Importantly, d‐MTxc in Aβ‐positive MCI was significantly lower than Aβ‐positive SCD. Stratification by median d‐MTxc revealed that high‐distraction‐cost individuals were predominantly SCD (66.7%), whereas low‐distraction‐cost individuals were mostly MCI (70%). Within the high‐d‐MTxc subgroup, Aβ‐positive SCD patients had significantly higher CRIW, CRIL, and CRIT than Aβ‐positive MCI patients. This pattern strongly supports our hypothesis that higher CR mitigates the clinical expression of Aβ pathology.

Neurobiologically, we identified 36 brain regions where Aβ SUVR positively correlated with both d‐MTxc and CRIW, including the insula, anterior cingulate, angular gyrus, and precuneus – regions overlapping with the DMN, salience, and executive control networks. The DMN is pivotal for attentional resource allocation; its aberrant activity impairs filtering of external interference during task execution.^17^

Furthermore, extending our ROI framework to FDG‐PET revealed a distinct mechanistic dissociation. CRIW was negatively correlated with FDG uptake in 11 core regions, including the precuneus and angular gyrus – key DMN hubs – consistent with the CR hypothesis. Conversely, the absence of significant correlations between d‐MTxc and regional FDG uptake indicates that the distraction cost does not merely reflect static metabolic degradation. Instead, d‐MTxc captures state‐dependent functional vulnerability – impaired compensatory neural recruitment under load – that remains hidden during resting‐state imaging. Together, these convergent findings establish that CRIW and d‐MTxc measure distinct neurobiological phenomena: long‐term tolerance to downstream neurodegeneration versus dynamic network failure under cognitive stress.

Metabolic network connectivity analysis further clarified disease progression: The Aβ‐positive MCI group showed weaker interregional metabolic connectivity compared with both SCD groups, particularly in temporal, parietal, and occipital lobes, reflecting disconnection syndrome secondary to Aβ‐induced pathology.^38^, ^39^

Several limitations should be acknowledged. First, this was a cross‐sectional observational study; longitudinal follow‐up is needed to confirm predictive utility. Second, CR was estimated via questionnaire rather than objective longitudinal data. Third, we lacked tau PET or other neurodegenerative biomarkers. Fourth, while subjective hearing impairment was excluded via clinical interviews and self/informant reports, we lacked formal audiometric assessments; future studies should incorporate objective audiometry to differentiate peripheral from central contributions. The within‐subject design partially mitigates stable sensory deficits, but Aβ pathology can also disrupt central auditory processing independent of peripheral hearing.^40^ The fixed quiet‐first task order may introduce order‐related confounds; future research should implement counterbalanced designs. Two observations argue against dominant practice effects: (1) overall accuracy declined from quiet to noisy conditions and (2) no significant improvement was observed between the first and second halves of the quiet condition. Nevertheless, we cannot quantify sequence‐induced biases. Finally, auditory and visual distractors impair visual task performance via partially distinct mechanisms,^41^ we lacked a visual‐distractor condition, and we cannot determine whether the observed sensitivity is specific to cross‐modal interference or reflects general attentional load. Future studies comparing multiple distractor modalities are needed; a purely visual distractor could simplify community deployment.

In conclusion, auditory distraction cost and occupational CR jointly identify Aβ positivity in SCD, with a combined model showing clinically useful diagnostic accuracy. Higher occupational reserve appears to compensate for Aβ‐associated vulnerability and delay progression to MCI. Neural correlates involve distributed early Alzheimer's disease‐vulnerable regions where Aβ deposition correlates with both metrics, while widespread metabolic network disruption characterizes the MCI transition. This brief, distraction‐modulated cognitive task combined with reserve assessment offers an accessible, non‐invasive strategy for preclinical Alzheimer's disease identification, warranting longitudinal validation.

## AUTHOR CONTRIBUTIONS

We confirm that all listed authors have contributed to this manuscript and that all significant contributors are included. Zhong Pei and Wenli Sheng conceived the study and supervised this work. Yating Wang and Yuanhong Chen contributed to data collection, data analysis, writing, and reviewing. Haifan Kong, Weineng Chen, Fengjuan Su, Yifan Zheng, Yingying Fang, Lishan Lin, Jiayi Zhou, Yucheng Li, Ya Zhang, and Anqi Huang contributed to data collection and writing. Michael F. Bergeron, James O. Clifford Jr, J Wesson Ashford, Xianbo Zhou, and Zhongliang Dai contributed to writing and reviewing. All authors reviewed and approved the final manuscript.

## CONFLICT OF INTEREST STATEMENT

The authors declare no conflicts of interest.Author disclosures are available in the Supporting Information.

## CONSENT STATEMENT

All participants or guardians voluntarily signed an informed consent.

## ETHICS APPROVAL AND CONSENT TO PARTICIPATE

This study was performed according to the Helsinki Declaration of 1975 and was approved by the ethical committee of the First Affiliated Hospital of Sun Yat‐sen University in Guangzhou (approval number: [2021]343‐1), Guangdong, China. All participants or guardians voluntarily signed an informed consent.

## Supporting information



Supporting Information

Supporting information
