# Supplementary material for: Unmasking preclinical Alzheimer's pathology: auditory distraction cost and occupational reserve jointly discriminate amyloid positivity in subjective cognitive decline
Source: Alzheimers Dement. 2026 Sep 21;22(9):e71874. doi: 10.1002/alz.71874 (PMC13594782; doi:10.1002/alz.71874)
Supplement: Supplementary file 2 [file ALZ-22-e71874-s002.docx]

**Supplementary Table 1. Clinical Data Comparison in MCI Aβ-negative vs. Aβ-positive Groups**

|  | **Aβ-negative (n=10)** | **Aβ-positive (n=37)** | **χ^2^/t/z** | ***P* value** |
| --- | --- | --- | --- | --- |
| Age, mean (SD), years | 67.8 (7.193) | 68.7 (8.356) | -0.311 | 0.757 |
| Sex, female, n (%) | 7 (70) | 20 (54.1) | 0.296 | 0.586 |
| MoCA, mean (SD) | 17.8 (2.781) | 17.27 (3.556) | 0.435 | 0.666 |
| Q-MTxc, mean (SD), % | 73.2 (7.131) | 73.3 (8.289) | -0.034 | 0.973 |
| Q-MTxrt, mean (SD), s | 1.486 (0.246) | 1.442 (0.297) | 0.432 | 0.667 |
| N-MTxc, median [P25, P75], % | 72 [61.5, 77] | 68 [61, 75] | -0.678 | 0.498 |
| N-MTxrt, median [P25, P75], s | 1.637 [1.313, 1.759] | 1.323 [1.140, 1.686] | -1.638 | 0.101 |
| d-MTxc, mean (SD), pp | 4.4 (10.532) | 5.95 (7.564) | -0.526 | 0.601 |
| CRIE, mean (SD) | 98.7 (11.823) | 107.22 (13.367) | -1.828 | 0.074 |
| CRIW, mean (SD) | 113.3 (26.924) | 113.22 (18.76) | 0.011 | 0.991 |
| CRIL, mean (SD) | 90.5 (10.554) | 90.51 (11.457) | -0.003 | 0.997 |
| CRIT, mean (SD) | 101 (16.997) | 104.76 (14.698) | -0.694 | 0.491 |

MoCA, Montreal Cognitive Assessment. Q-MTxc, accuracy rate of MemTrax in a quiet environment. Q-MTxrt, reaction time of MemTrax in a quiet environment. N-MTxc, accuracy rate of MemTrax in a noisy environment. N-MTxrt, reaction time of MemTrax in a noisy environment. d-MTxc, and the distraction cost representing the difference in MemTrax accuracy between the quiet and noisy environments. pp, percentage point. CRIE, education scores of cognitive reserve index questionnaire. CRIW, work scores of cognitive reserve index questionnaire. CRIL, leisure activity scores of cognitive reserve index questionnaire. CRIT, total scores of cognitive reserve index questionnaire. Note: χ² values are reported for categorical variables (Chi-square test); t values are reported for normally distributed continuous variables (independent samples t-test); z values are reported for non-normally distributed continuous variables (Mann-Whitney U test).

**Supplementary Figure 1.** **Heatmap of regional correlations between Aβ SUVR and d-MTxc/CRIW scores.** Note: The 40 brain regions displayed exhibited significant positive correlations with both indices at an uncorrected threshold of P < 0.05. Numbers within cells represent correlation coefficients (Pearson’s r or Spearman’s r_s_, determined based on data normality). *P < 0.05, **P < 0.01, ***P < 0.001.

**Supplementary Table 2. Information on brain regions exhibiting significant positive correlations of regional Aβ deposition with both d-MTxc and CRIW scores.**

|  |  |  | **MNI Coordinates (mm)** | | | |
| --- | --- | --- | --- | --- | --- | --- |
| **Regions** | **Regions** | **abbr.** | **x(mm)** | **y(mm)** | **z(mm)** | |
| Frontal_Inf_Tri_L | Inferior frontal gyrus, triangular part | IFGtriang.L | -45.58 | 29.91 | 13.99 | |
| Frontal_Inf_Tri_R | Inferior frontal gyrus, triangular part | IFGtriang.R | 50.33 | 30.16 | 14.17 | |
| Frontal_Inf_Orb_R | Inferior frontal gyrus, orbital part | ORBinf.R | 41.22 | 32.23 | -11.91 | |
| Rolandic_Oper_L | Rolandic operculum | ROL.L | -47.16 | -8.48 | 13.95 | |
| Olfactory_L | Olfactory cortex | OLF.L | -8.06 | 15.05 | -11.46 | |
| Olfactory_R | Olfactory cortex | OLF.R | 10.43 | 15.91 | -11.26 | |
| Rectus_L | Gyrus rectus | REC.L | -5.08 | 37.07 | -18.14 | |
| Rectus_R | Gyrus rectus | REC.R | 8.35 | 35.64 | -18.04 | |
| Insula_L | Insula | INS.L | -35.13 | 6.65 | 3.44 | |
| Insula_R | Insula | INS.R | 39.02 | 6.25 | 2.08 | |
| Cingulum_Ant_L | Anterior cingulate and paracingulate gyri | ACG.L | -4.04 | 35.40 | 13.95 | |
| Cingulum_Ant_R | Anterior cingulate and paracingulate gyri | ACG.R | 8.46 | 37.01 | 15.84 | |
| Cingulum_Mid_L | Median cingulate and paracingulate gyri | DCG.L | -5.48 | -14.92 | 41.57 | |
| Cingulum_Mid_R | Median cingulate and paracingulate gyri | DCG.R | 8.02 | -8.83 | 39.79 | |
| Amygdala_L | Amygdala | AMYG.L | -23.27 | -0.67 | -17.14 | |
| Calcarine_L | Calcarine fissure and surrounding cortex | CAL.L | -7.14 | -78.67 | 6.44 | |
| Cuneus_L | Cuneus | CUN.L | -5.93 | -80.13 | 27.22 | |
| Lingual_L | Lingual gyrus | LING.L | -14.62 | -67.56 | -4.63 | |
| Occipital_Inf_L | Inferior occipital gyrus | IOG.L | -36.36 | -78.29 | -7.84 | |
| Parietal_Inf_L | Inferior parietal, but supramarginal and angular gyri | IPL.L | -42.80 | -45.82 | 46.74 | |
| SupraMarginal_L | Supramarginal gyrus | SMG.L | -55.79 | -33.64 | 30.45 | |
| SupraMarginal_R | Supramarginal gyrus | SMG.R | 57.61 | -31.50 | 34.48 | |
| Angular_L | Angular gyrus | ANG.L | -44.14 | -60.82 | 35.59 | |
| Angular_R | Angular gyrus | ANG.R | 45.51 | -59.98 | 38.63 | |
| Precuneus_L | Precuneus | PCUN.L | -7.24 | -56.07 | 48.01 | |
| Precuneus_R | Precuneus | PCUN.R | 9.98 | -56.05 | 43.77 | |
| Putamen_L | Lenticular nucleus, putamen | PUT.L | -23.91 | 3.86 | 2.40 | |
| Putamen_R | Lenticular nucleus, putamen | PUT.R | 27.78 | 4.91 | 2.46 | |
| Pallidum_L | Lenticular nucleus, pallidum | PAL.L | -17.75 | -0.03 | 0.21 | |
| Pallidum_R | Lenticular nucleus, pallidum | PAL.R | 21.20 | 0.18 | 0.23 | |
| Heschl_L | Heschl gyrus | HES.L | -41.99 | -18.88 | 9.98 | |
| Heschl_R | Heschl gyrus | HES.R | 45.86 | -17.15 | 10.41 | |
| Temporal_Sup_L | Superior temporal gyrus | STG.L | -53.16 | -20.68 | 7.13 | |
| Temporal_Sup_R | Superior temporal gyrus | STG.R | 58.15 | -21.78 | 6.80 | |
| Temporal_Mid_L | Middle temporal gyrus | MTG.L | -55.52 | -33.80 | -2.20 | |
| Temporal_Mid_R | Middle temporal gyrus | MTG.R | 57.47 | -37.23 | -1.47 |  |

**Supplementary Figure 2. Heatmap of regional correlations between FDG SUVR and CRIW scores.** Note: The 19 brain regions displayed exhibited significant negative correlations with CRIW at an uncorrected threshold of P < 0.05. Numbers within cells represent correlation coefficients (Pearson’s r or Spearman’s r_s_, determined based on data normality). *P < 0.05, **P < 0.01, ***P < 0.001.

**Supplementary Table 3. Information on brain regions exhibiting significant negative correlations of regional FDG metabolism with CRIW scores.**

|  |  |  | **MNI Coordinates (mm)** | | |
| --- | --- | --- | --- | --- | --- |
| **Regions** | **Regions** | **abbr.** | **x(mm)** | **y(mm)** | **z(mm)** |
| Calcarine_L | Calcarine fissure and surrounding cortex | CAL.L | -7.14 | -78.67 | 6.44 |
| Calcarine_R | Calcarine fissure and surrounding cortex | CAL.R | 15.99 | -73.15 | 9.40 |
| Cuneus_R | Cuneus | CUN.R | 13.51 | -79.36 | 28.23 |
| Lingual_L | Lingual gyrus | LING.L | -14.62 | -67.56 | -4.63 |
| Lingual_R | Lingual gyrus | LING.R | 16.29 | -66.93 | -3.87 |
| Occipital_Inf_L | Inferior occipital gyrus | IOG.L | -36.36 | -78.29 | -7.84 |
| Occipital_Inf_R | Inferior occipital gyrus | IOG.R | 38.16 | -81.99 | -7.61 |
| Angular_L | Angular gyrus | ANG.L | -44.14 | -60.82 | 35.59 |
| Angular_R | Angular gyrus | ANG.R | 45.51 | -59.98 | 38.63 |
| Precuneus_L | Precuneus | PCUN.L | -7.24 | -56.07 | 48.01 |
| Precuneus_R | Precuneus | PCUN.R | 9.98 | -56.05 | 43.77 |
